# Early life nociceptive stimulus and fentanyl exposure increase hippocampal neurogenesis and anxiety but do not affect spatial learning and memory

**DOI:** 10.3389/fnins.2022.988096

**Published:** 2022-09-29

**Authors:** Debora da Silva Bandeira Rêgo, Clivandir S. Silva, Luiz Eugenio A. M. Mello, Ana Teresa Figueiredo Stochero Leslie

**Affiliations:** ^1^Laboratório de Neurobiologia, Departamento de Fisiologia, Universidade Federal de São Paulo, São Paulo, Brazil; ^2^Instituto D’Or de Pesquisa e Ensino, IDOr, São Paulo, Brazil; ^3^Departmento de Pediatria, Universidade Federal de São Paulo, São Paulo, Brazil

**Keywords:** animal, neonatal, pain, opioid, neurogenesis, spatial learning, anxiety

## Abstract

This study aimed to determine whether preemptive fentanyl administration in neonatal rats reduces the impact of a nociceptive stimulus initiated during the first day of life (P1) on hippocampal neurogenesis, behavior, and learning. At P1, Wistar rat pups received either a subcutaneous injection of fentanyl (F) before intraplantar injection of complete Freund’s adjuvant (CFA) (CFA + F group), an isolated injection of CFA (CFA group), or subcutaneous injection of fentanyl without CFA injection (F). Control animals received saline injections using the same route and volume as the treatment groups. Hippocampal neurogenesis was evaluated by 5′ –bromo-2′-deoxyuridine (BrdU) staining on P10 and P39 to assess neuronal proliferation and survival, respectively. Anxiety behavior in adulthood was assessed using an open field test (OF) and an elevated plus maze test (EPM). Spatial memory was assessed on a Morris water maze test (MWM), where the animals were trained for seven days, beginning on P81, and the probe trial was performed to evaluate memory retention. Although the CFA + F group showed an increased number of proliferative cells on P10, this finding did not persist on P39. The CFA + F group spent more time in the closed arms in the EPM, revealing more anxious behavior, although the early noxious experience, both with and without fentanyl, did not alter neurogenesis in adolescence and learning in adulthood. This study highlights that the impact of pain in early life pain combined with fentanyl on hippocampal neurogenesis on P10 did not persist on P39. In addition, this combined intervention during the first week of life was associated with higher anxiety levels.

## Introduction

Every year, an estimated 15 million babies are born preterm worldwide (Blencowe et al., 2012). Concerns about the long-term neurodevelopmental outcomes associated with the complications of prematurity have been raised for the past 30 years. The life-saving care premature infants receive in neonatal intensive care units (NICU) involves multiple invasive procedures each day, which are often painful and may be accompanied by inflammation and tissue injury during a period of structural brain organization and establishment of functional networks (Ranger and Grunau, 2014). Several studies have shown poor pain treatment in the NICU (Anand, 2007). The EPIPPAIN study (Epidemiology of Procedural Pain in Neonates) (Carbajal et al., 2008) evaluated the number of painful or stressful procedures between September 2005 and January 2006 from the first 14 days of admission and were prospectively collected for a 6-week period from 430 neonates admitted to tertiary care centers in the Paris region. Each infant experienced a median of 115 procedures and 16 procedures per day of hospitalization, whereas the most common was heelstick.

Despite being immature at birth, the nociceptive pathways are functioning and, consequently, noxious procedures produce physiological and behavioral nociceptive responses (Beggs, 2015). Neonatal pain has been shown to have long-lasting consequences on the nociceptive neural pathways (Schwaller and Fitzgerald, 2014); therefore, pain exposure during the first weeks/months of life is associated with altered long-term neurodevelopmental and behavioral outcomes (Grunau, 2013; Williams and Lascelles, 2020). Preterm infants show a thinner cortex in multiple brain regions at school age, which may be related to neonatal pain (Ranger et al., 2013). In addition, the stress related to pain experienced during neonatal life has been associated with lower cognitive and motor function at 8 and 18 months corrected age and higher internalizing behaviors at the age of 18 months and 7 years (Vinall and Grunau, 2014).

Despite the substantial evidence for the long-term influence of untreated pain on the central nervous system (CNS) (Ranger and Grunau, 2014; Schwaller and Fitzgerald, 2014), data regarding the benefits of the use of opioids, which are considered the gold standard analgesic drugs for moderate to severe painful procedures in the NICU, is contradictory (Laprairie et al., 2008). Clinical data suggest that fentanyl reduces behavioral responses in mechanically ventilated infants (Guinsburg et al., 1998) and decreases mortality (Anand and Hickey, 1992). However, opioid exposure has also been reported to decrease dendritic spine density and neurogenesis and alter synaptic transmission in the hippocampus in adult life (Corrigall, 1983), leading to neurobehavioral deficits (McPherson et al., 2007; Boasen et al., 2009). Interestingly, Kahn et al. found that narcotic administration occurred more frequently among the smallest infants (<750 g, 22%; 750–999 g, 13%; and 1,000–1,499 g, 8%) (Kahn et al., 1998).

Strong evidence has suggested that neurogenesis continues throughout adulthood in the hippocampal dentate gyrus (Christie and Cameron, 2006) and it is well demonstrated that adverse events may downregulate this process, such as stress (Gould and Tanapat, 1999). It has been demonstrated that chronic exposure to morphine decreases neurogenesis by 42% in adult rats (Eisch et al., 2000). Whether neonatal exposure to opioids alters hippocampal neurogenesis remains under some debate. On the other hand, antidepressant treatment increases adult neurogenesis (Malberg et al., 2000; Manev et al., 2001), as well as environmental enrichment (Kempermann et al., 1997; van Praag et al., 1999a) and voluntary exercise (van Praag et al., 1999a; Olson et al., 2006).

Rats are born at a stage of brain maturation comparable to that of humans at the third trimester, which makes them appropriate for studying the effects of neonatal pain on early developing brains (Romijn et al., 1991; Simonato, 1996; Eisch et al., 2000). Various rodent models have been used to study the long-term effects of neonatal injury on the central nervous system (Xia et al., 2020; Steinbauer et al., 2022), including surgical incision (Walker et al., 2009), skin wounds (Beggs et al., 2012), needles (Anand et al., 1999), and inflammation (Hohmann et al., 2005; Leslie et al., 2011).

The involvement of the continuous generation of new neurons in the functioning of hippocampus-dependent learning and memory formation has been supported by growing evidence (Scoville and Milner, 1957; Squire, 1982; Moscovitch et al., 2005). It has been suggested that synaptic plasticity in the hippocampus may support acquisition and memory retention (Martin et al., 2000; Lamprecht and LeDoux, 2004). As mentioned before, several factors and conditions have been shown to affect the number of new neurons in the dentate gyrus (DG) of adult vertebrates. However, the response of neonatal neurogenesis to different stimuli has not been completely elucidated. Therefore, this study hypothesizes that exposure to pain in neonatal rats modifies hippocampal neurogenesis, leading to alterations in anxiety behavior and learning in adulthood, and that this impact could be attenuated by the preemptive treatment of these animals with fentanyl.

## Materials and methods

### Animals

Time-pregnant Wistar rats were obtained on the 14th day of gestation from the Center for Development of Animal Models of the *Universidade Federal de São Paulo* and housed individually. Animals were housed in plastic home cages and were maintained in a dark/light cycle of 12 h, with the light cycle beginning at 7:00 am, and a constant temperature (23 ± 2°C) and *ad libitum* access to food and water. The first day of life was designated as P1, and rat pups of both sexes were utilized. All experiments were approved by the Institutional Ethics Committee for Animal Research of *Universidade Federal de São Paulo* (protocol number 0300/12) and were conducted in accordance with the guidelines of the International Association for the Study of Pain (Zimmermann, 1983). All efforts were made to minimize the number of animals used and their discomfort. Treatment of all litters was performed between 7:00 and 9:00 am.

### Early life manipulation

**FIGURE 1:**
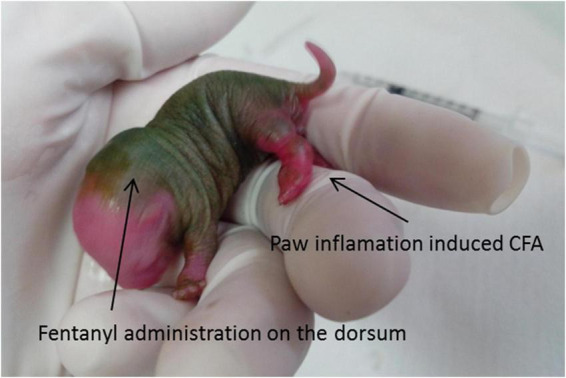
CFA injection. Note the robust inflammation in the left paw. The fentanyl administration on the dorsum was subcutaneous. CFA, inflammatory agent complete Freund’s adjuvant.

**FIGURE 2:**
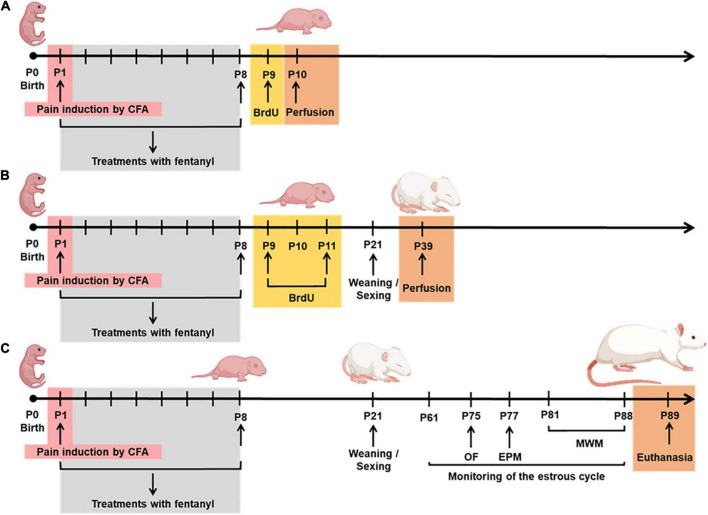
Experimental protocol from P1-P89. **(A)** Cell proliferation on P10; **(B)** neurogenesis on P39; **(C)** behavior and learning in adulthood. P, postnatal; CFA, inflammatory agent complete Freund’s adjuvant; BrdU, 5-bromo-2-deoxyuridine; OF, open field; EPM, elevated plus maze; MWM, Morris water maze.

Each cage contained a dam and 10 rat pups (we tried to balance the number of females and males) that were randomly assigned to each experimental group. All experiments were initiated on the first day of life (P1), and the pups remained with their dams until P21, when they were weaned and separated into groups of four to five pups per cage, putting together littermates of the same sex. The treatment groups were as follows: (1) Naïve, rats that received no treatment; (2) Sham 1, rats that received an equivolume of sterile normal saline into the left hindpaw on P1; (3) Sham 2, rats that received an equivolume of sterile normal saline into the left hindpaw on P1, followed by a daily subcutaneous mid-scapular injection of 0.9% saline injection from P1 to P8; (4) complete Freund’s adjuvant (CFA; 25 μL, Sigma, Saint Louis, MI) (pain group), rats that received a single intraplantar injection of the inflammatory agent (Duric and McCarson, 2006) into the left hindpaw on P1, as previously reported by Leslie et al. (2011) (Figure 1); (5) CFA + F (analgesia group), rats that received fentanyl sulfate (100 μg/kg, s.c., in the mid-scapular area, *Cristalia Ltda, São Paulo*, Brazil) 30 min before CFA injection on P1 and subsequent doses from P2 to P8; (6) F, rats that received only subcutaneous fentanyl on consecutive postnatal days P1–P8 to evaluate the neurotoxic effects of fentanyl itself. As shown in Figure 2, rats of all experimental groups were allocated to three subsets of animals to assess neurogenesis at P9 and P39 and behavior and learning in adulthood. A total of 253 animals were evaluated for all procedures, and 12 pups died during the neonatal manipulation (4.7% mortality rate).

### Neurogenesis

To determine the effects of a noxious stimulus initiated on the first day of life, with or without fentanyl administration during the first week of life, immunohistochemical analysis was performed to identify cells at different stages of neurogenesis (proliferation, survival, and differentiation). 5-bromo-2-deoxyuridine (BrdU) is well known to incorporate during the S phase of the cell cycle, when the DNA is replicated. Thus, any cell undergoing cell division incorporates BrdU, and the cells resulting from this first cell will also have BrdU in their DNA. We injected BrdU at two different time points: (1) to assess the immediate impact of adverse neonatal experience on cell proliferation (Figure 2A); (2) to assess the cell survival during a 4-week period after the neonatal manipulation (Figure 2B).

#### Proliferation

To detect proliferating cells, 48 pups (eight per group, four of each sex) were treated with BrdU (Sigma; Saint Louis, MO, USA), two doses of 100 mg/kg, freshly prepared, dissolved in phosphate buffer solution [PBS] in a dilution of 10 mg/mL, i.*p*., every 12 h) (Christie and Cameron, 2006). Twenty-four hours after the last BrdU injection, on P10, the animals were deeply anesthetized with sodium pentobarbital (50 mg/kg, i.*p*., *Cristalia*, São Paulo, Brazil) and perfused transcardially through the left ventricle with 0.9% normal saline solution followed by ice-cold 4% paraformaldehyde in 0.1 M PBS (pH 7.4). Brains were removed, post-fixed overnight in 4% paraformaldehyde, and cryoprotected in 30% sucrose for 24 h. Coronal brain sections (30 μm thickness) were obtained across the entire dorsal-ventral extension of the DG using a Leica CM1850 cryostat (Nussloch, Germany) and stored at –20°C in cryoprotectant. Six sections obtained at regular intervals of approximately 40 μm were selected from each animal. For BrdU immunohistochemistry, sections were first rinsed with PBS, treated with 2 N HCl at 37°C for 30 min, and then incubated for 15 min in 1% hydrogen peroxide to eliminate endogenous peroxidases. The sections were incubated overnight with the primary antibody (monoclonal rat anti-BrdU, 1:500; Abcam, Cambridge, United Kingdom) at room temperature and the secondary antibody (biotinylated goat anti-rat; 1:200, Jackson Immuno Research, West Grove, PA, USA) at room temperature for 2 h. Antibodies were diluted in a blocking solution containing 5% goat serum and 0.1% Triton X-100 dissolved in PBS. BrdU-positive cells were visualized using the avidin–biotin-peroxidase complex (ABC, Vector Laboratories Inc., CA, USA), followed by treatment with diaminobenzidine (DAB, Sigma, Saint Louis, MO, USA). Slides were dehydrated, and coverslipped with Permount (Sigma, Saint Louis, MO, USA). Coded slides were evaluated for BrdU-positive cells, which were manually counted from the subgranular zone (SGZ, at the junction between the granule cell layer and the hilus) using a microscope (Nikon Eclipse 50i) with a 40× magnification. The code was opened only once all the slides were analyzed.

#### Cell survival

To evaluate the survival of newly generated cells in the DG, 60 animals (10 per group, five of each sex) received a total of four doses of BrdU every 12 h from P9, for 2 consecutive days (P9–P10) (Figure 2B). On P39 (4 weeks after BrdU injection), the animals were perfused and the total number of BrdU-positive cells was counted in the subgranular zone, using the same procedures that were performed to assess cell proliferation (van Praag et al., 1999b).

#### Cell differentiation

To estimate the phenotype of the newborn cells, colocalization of BrdU and the neuronal nuclear antigen marker (NeuN) and BrdU and glial fibrillary acidic protein (GFAP, an astrocyte marker) were assessed (Christie and Cameron, 2006). Double-immunostaining was performed with immunofluorescence. Sections were incubated overnight at 4*^o^*C with the rat monoclonal anti-BrdU antibody (1:500, Abcam, Cambridge, United Kingdom) and polyclonal goat anti-GFAP antibody (1:500, Abcam, Cambridge, United Kingdom). For the immunohistochemistry for BrdU and NeuN, the sections were incubated in the primary antibody overnight with anti-BrdU and anti-NeuN (rabbit, 1:500, Millipore, Germany). BrdU/NeuN colocalization was calculated by dividing the number of double-labeled cells by the number of BrdU-positive cells (Malheiros et al., 2014). The rater was blind to the treatment. BrdU^+^/NeuN^+^ cells in the subgranular zone were counted in a one-in-six series of sections (120 μm apart) throughout the dorsal hippocampus using a Nikon Eclipse 50i (Nikon DXM 1200 camera and ACT-1 software), with a 40× objective to confirm colocalization. Six DG per animal were analyzed. Data are presented as the total number of BrdU^+^/NeuN^+^ cells per treatment group. We assessed the coexpression of BrdU and GFAP by determining the amount of signal overlap using a fluorescence microscope Nikon Eclipse 50i, with a 40× objective. At least six equidistant sections, per dentate gyrus of each animal. Percentage of coexpression was considered the ratio of counted cells coexpressing BrdU and GFAP to the total counted the number of BrdU labeled cells multiplied by 100.

### Adult behavior and learning

#### Estrous cycle

Females were monitored by vaginal smears between 9:00 and 10:00 am daily from P61 (Figure 2C). The vaginal smears were collected each day to assess the stage of estrous, as previously described (Hodes and Shors, 2005). Distilled water (10 μL) was pipetted inside the vagina to pick up cells. The cells were placed onto slides and stages of the estrous cycle were verified by visualization under a Nikon Eclipse E400 light microscope with 10 × magnification. For behavioral tests, a total of 133 animals were studied (at least 10 per sex for each group).

#### Open field

The open field (OF) apparatus consisted of a square arena (75 cm × 75 cm) with 50-cm high walls. The “central area” of the arena was defined as a centrally positioned circle. The OF behavior of the rats was recorded on P75 and analyzed with an EthoVision System XT 8 (Noldus Information Technology, Netherlands). Animals were exposed to the OF for 5 min on P75 (Figure 2C). The experiment started immediately after the animals were placed in the central area. We analyzed the time spent in each zone (outer and inner) and the total distance moved. To measure anxiety behavior, we analyzed the time that the animal avoided the inner zone of the arena. The OF apparatus was cleaned with 5% alcohol solution and dried with paper towels between each test.

#### Elevated plus maze

The elevated plus maze test (EPM), which is based on the innate fear rodents have for open and elevated spaces (Pellow et al., 1985), was performed 48 h after the OF test to evaluate anxiety-like behavior, and animals were observed in the EPM on P77 (Figure 2C). The apparatus was raised 50 cm above the floor level and consisted of four arms (10 cm × 50 cm): two enclosed arms opposed perpendicularly by the two arms. All of the arms had a central intersection (10 cm × 10 cm). At the beginning of the test, each rat was placed at the central intersection facing the open arm. Time percentage of entries and time spent in the open arms were measured for 5 min in the EPM.

#### Morris water maze

To test the integrity of the hippocampus in adulthood, rats underwent training in the Morris water maze (MWM) on P81 (Figure 2C). The animals were trained to find a hidden submerged platform (8 cm in diameter) in a circular pool (200 cm in diameter, 25 cm deep, 40 cm high) filled with tap water at 26°C for seven consecutive days with four trials per day. Each rat was given 60 s to find the hidden platform in each trial. Trials began when the rats were released from one of five points (northeast, east, southeast, south, and southwest) equally spaced around the perimeter of the pool with their heads facing the pool wall. Each release point was used an equal number of times per day, and the sequence of release points was randomized within and across days. Trials ended when rats reached the platform. If the rat failed to find the platform within 60 s, the trial was terminated, and the animal was guided and placed to the platform by the investigator and left there for 15 s. The latency was calculated as the average time to find the platform in the four trials performed each day. The inter-trial interval was around 5 min. Performance during training was measured by determining the latency to reach the platform. The rat behavior was monitored with a video camera connected to a computer. For data analysis, the pool was divided into four quadrants (target quadrant, left, right, and the opposite of the target quadrant). After seven days of training (measured by the latency to find the platform), the platform was removed so the probe trial could be performed to check memory retention in the animal. We analyzed the learning curve of the animals over the seven days of training through the duration of time in the arena. Once the animals learned, the time spent finding the location of the platform tended to decrease. Also, in the probe trial, we looked at latency in each of the four quadrants (target, opposite, right and left), the speed of the swim, and the total distance measured using Etho-Vision XT 7.0 software (Noldus Information Technology Inc., Leesburg, VA, USA). On P89, the animals were sacrificed after a lethal dose of pentobarbital followed by decapitation. Brain mass was weighted on a *Bioprecisa* scale (JA3003N), and cerebellar mass was measured using a Shimadzu scale (AY220). The body mass was weighted on a Toledo scale (9094-II) on P1, P9, and P89.

### Statistical analysis

Data are expressed as mean ± SEM. Data analysis was performed using a Kolmogorov–Smirnov test to verify the distribution. Significant main effects of neonatal treatment and sex were assessed using a two-way analysis of variance (ANOVA). A Bonferroni *post hoc* test was used to determine differences in relation to treatment and/or sex. Statistica 12 and SPSS 20.0 software were used for statistical analysis. The graphs were performed using GraphPad Prism 5. Statistical significance was set at *P* < 0.05.

## Results

### Neurogenesis

#### Cell proliferation

**FIGURE 3:**
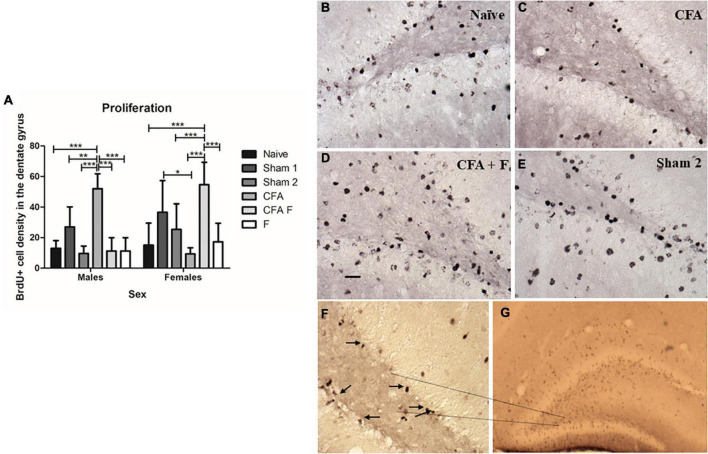
Cell proliferation in the dentate gyrus on P10. **(A)** There was an interaction between Group*Sex [*F*_(5,44)_ = 11.963, *p* = 0.00]. Among females, the CFA + F group showed the highest number of BrdU-labeled cells in the dentate gyrus, whereas the CFA group showed the lowest number of BrdU-labeled cells. The CFA group demonstrated a decreased number of BrdU-labeled cells when compared to Sham 1 (*p* = 0.02) and CFA + F (*p* = 0.00) groups. The CFA + F group showed higher number of BrdU-labeled cells when compared to Naïve (*p* = 0.00), Sham 2 (*p* = 0.03), CFA (*p* = 0.00) and F (*p* = 0.00). Oppositely, among males, the CFA group demonstrated the highest number of BrdU-labeled cells in the dentate gyrus, when compared to Naïve (*p* = 0.00), Sham 1 (*p* = 0.01), Sham 2 (*p* = 0.00), CFA + F (*p* = 0.00) and F (*p* = 0.00). **(B–E)** examples of BrdU-labeled cells in the dentate gyrus. **(F)** Subgranular zone of dentate gyrus, 40× magnification, the arrows demonstrate the BrdU-labeled cells. **(G)** BrdU-labeled cells in the dentate gyrus, 4× magnification. Scale bar: 25 μm. *p < 0.05; **p < 0.01; ***p < 0.001.

At P10, we found a significant main effect for group in the number of BrdU-labeled cells in the DG [*F*_(5,44)_ = 4.862, *p* = 0.001] (Figure 3A). The animals that received the noxious stimulus induced by CFA injection along with administration of fentanyl (CFA + F) displayed a significantly higher number of BrdU-labeled cells in the DG of the hippocampus (Figure 3A), in comparison with other treatment groups. No differences were observed between sexes [*F*_(1,44)_ = 2.718, *p* = 0.106]. There was an interaction between Group*Sex [*F*_(5,44)_ = 11.963, *p* = 0.00], where the males of CFA group showed an increased number of BrdU-labeled cells when compared to Naïve (*p* = 0.00), Sham 1 (*p* = 0.01), Sham 2 (*p* = 0.00), CFA + F (*p* = 0.00) and F (*p* = 0.00) (Figure 3A). On the other hand, among female animals, the pain group (CFA) demonstrated a decreased number of BrdU-labeled cells when compared to Sham 1 (*p* = 0.02) and CFA + F (*p* = 0.00) groups. The CFA + F group showed the highest number of BrdU-labeled cells when compared to Naïve (*p* = 0.00), Sham 2 (*p* = 0.03), CFA (*p* = 0.00) and F (*p* = 0.00) (Figure 3A). Figures 3B–G illustrate the morphology of BrdU-labeled cells, characterized by darkly stained with an irregular shape, localized within the subgranular zone (the border between the granule cell layer and the hilus).

### Cell survival

**FIGURE 4:**
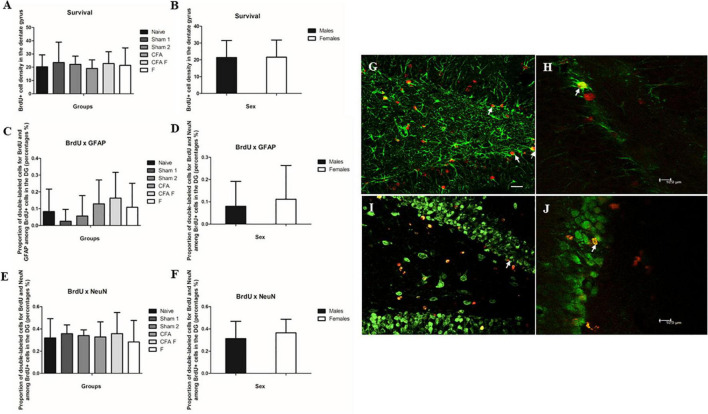
Cell survival and cell differentiation in the dentate gyrus. **(A)** No significant differences were found in BrdU-labeled cells in the dentate gyrus among the treatment groups. **(B)** No significant differences were found in BrdU-labeled cells in the dentate gyrus between the sexes. **(C)** No significant differences were found for double-labeled cells GFAP^+^/BrdU^+^ in the dentate gyrus among the treatment groups. **(D)** No significant differences were found for double-labeled cells GFAP^+^/BrdU^+^ in the dentate gyrus between the sexes. **(E)** No significant differences were found for double-labeled cells NeuN^+^/BrdU^+^ in the dentate gyrus among the treatment groups. **(F)** No significant differences were found for double-labeled cells NeuN^+^/BrdU^+^ in the dentate gyrus between the sexes. **(G)** Fluorescence microscopy, 40× magnification. The arrows show double-labeled cells by BrdU and GFAP (BrdU red and GFAP green). **(H)** Confocal image. The arrow shows a double-labeled cell by BrdU and GFAP (BrdU red and GFAP green). **(I)** Fluorescence microscopy, 40× magnification. The arrow shows a double-labeled cell by BrdU and NeuN (BrdU red and NeuN green). **(J)** Confocal image. The arrow shows a double-labeled cell by BrdU and NeuN (BrdU red and NeuN green). Scale bar for panels **(G)** and **(I)** 25 μm.

To evaluate the number of subgranular cell survival in the dentate gyrus, we found that number of BrdU-labeled cells in P39 did not show differences among the treatment groups [*F*_(5,55)_ = 0.317, *p* = 0.901] (Figure 4A), between sexes [*F*_(1,55)_ = 0.003, *p* = 0.959] (Figure 4B), nor an interaction between Group*Sex [*F*_(5,55)_ = 0.425, *p* = 0.829].

#### Cell differentiation

To determine the phenotype of BrdU-labeled cells, we assessed the percentage of BrdU-labeled cells that became astrocytes (GFAP) (Figures 4C,D,G,H) or neurons (NeuN) (Figures 4E,F,I,J). No differences were observed in the number of BrdU^+^/GFAP^+^ cells among the groups [*F*_(5,46)_ = 1.099, *p* = 0.374] (Figure 4C) and between sexes [*F*_(5,46)_ = 0.289, *p* = 0.594] (Figure 4D). We did not find an interaction between Group*Sex [*F*_(5,46)_ = 1.239, *p* = 0.306].

No differences were observed in the number of BrdU^+^/NeuN^+^ cells among the groups [*F*_(5,54)_ = 1.599, *p* = 0.176] (Figure 4E) and between sexes [*F*_(5,54)_ = 2.197, *p* = 0.144] (Figure 4F). We detected an interaction between Group*Sex [*F*_(5,54)_ = 3.114, *p* = 0.015], which Bonferroni test did not confirm this finding.

### Adult behavior and learning

#### Estrous cycle

There was no influence of the estrous cycle on all behavior variables (Supplementary Table 1). We evaluated the following phases: proestrus, estrus, metestrus, and diestrus (Supplementary Table 1).

#### Open field

**FIGURE 5:**
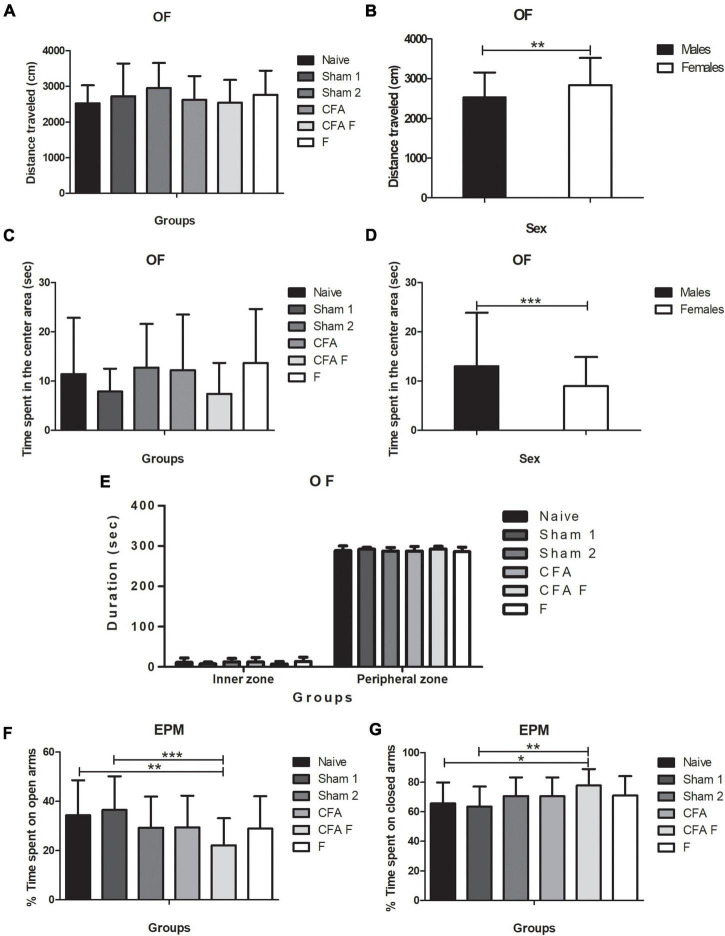
**(A)** No differences in the distance traveled were observed among the groups. **(B)** Females traveled a longer distance than males (*p* = 0.01). **(C)** No significant effect was observed for the duration in the inner zone among groups (*p* = 0.11). **(D)** The males spent more time than females in the central area (*p* = 0.01). **(E)** No significant differences in the duration (sec) in the inner and outer zones were observed. **(F)** CFA + F group spent less time in the open arms in the EPM when compared to Naïve (*p* = 0.03) and Sham 1 (*p* = 0.005). **(G)** CFA + F spent more time in the closed arms in the EPM when compared to Naïve (*p* = 0.028) and Sham 1 (*p* = 0.005). OF, open field; EPM, elevated plus maze (*p < 0.05; **p < 0.01; ***p < 0.001).

We investigated the locomotor and exploratory activities of rats as a measure of anxiety-like behavior. The groups showed no differences for distance traveled [*F*_(5,121)_ = 1.063; *p* = 0.38], illustrated in Figure 5A, although a main effect of sex was detected for the total distance traveled [*F*_(1,121)_ = 6.7483; *p* = 0.01] (Figure 5B). Females (2834 ± 687 cm) traveled longer distances than males (2527.6 ± 629 cm), as shown in Figure 5B. There was no interaction between Group*Sex for distance traveled [*F*_(5,121)_ = 0.856; *p* = 0.51]. No significant effect was observed for duration in the center area among the groups [*F*_(5,121)_ = 1.814; *p* = 0,11] (Figure 5C), males spent more time in the center [*F*_(1,121)_ = 6.2889; *p* = 0.01] (Figure 5D), indicating less anxious behavior. No interaction between Group*Sex [*F*_(5,121)_ = 0.113; *p* = 0.989] was detected. Regarding the duration spent in the peripheral zone, no differences were found among the groups [*F*_(5,121)_ = 1.911; *p* = 0.09] (Figure 5E). Females stayed longer in the peripheral zone [*F*_(1,121)_ = 5.776; *p* = 0.01] but there was no interaction between Group*Sex [*F*_(5,121)_ = 0.086; *p* = 0.994].

#### Elevated plus maze

A main effect of group was detected [*F*_(5,121)_ = 3.354; *p* = 0.007], wherein the naïve (*p* = 0.03) and sham 1 (*p* = 0.005) rats spent more time in the open arms than the CFA + F group (Figure 5F); this finding indicated more anxious behavior among the animals subjected to nociceptive stimulus combined with fentanyl. However, no significant difference was observed between sexes [*F*_(1,121)_ = 0.448; *p* = 0.504], nor an interaction between Group*Sex [*F*_(5,121)_ = 0.953; *p* = 0.449]. For the percentage of time spent on closed arms, a difference was found among the groups [*F*_(5,121)_ = 3.354; *p* = 0.007] (Figure 5G), where the Naïve (*p* = 0.028) and Sham 1 (*p* = 0.005) groups spent shorter time in the closed arms when compared to CFA + F. No differences were detected between males and females [*F*_(1,121)_ = 0.448; *p* = 0.504], nor an interaction for Group*Sex [*F*_(5,121)_ = 0.953; *p* = 0.449]. For the percentage of time spent on open arms, no significant differences were observed among the groups [*F*_(5,121)_ = 0.229; *p* = 0.949] and between sexes [*F*_(1,121)_ = 0.249; *p* = 0.619]. Also, there was no interaction between Group*Sex [*F*_(5,121)_ = 0.657; *p* = 0.657]. Entries in the closed arms were not significantly different among the groups [*F*_(5,121)_ = 0.229; *p* = 0.949], between females and males [*F*_(1,121)_ = 0.249; *p* = 0.619]. No interaction between Group*Sex [*F*_(1,121)_ = 0.249; *p* = 0.619] was found.

#### Morris water maze

**FIGURE 6:**
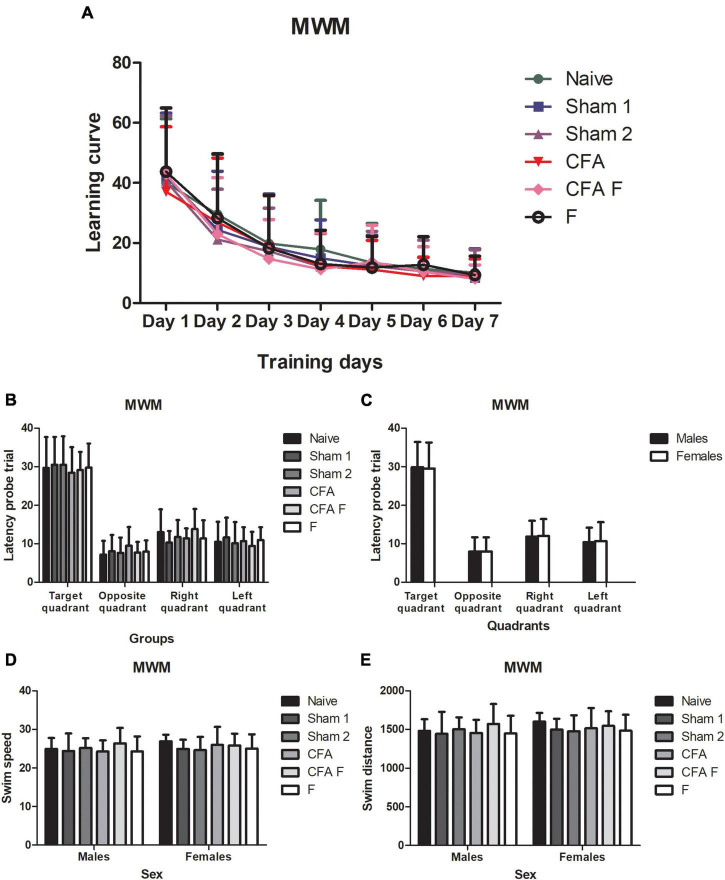
**(A)** All animals demonstrated a clear reduction in latency equally over seven days. **(B)** The groups showed the same preference for target quadrant. **(C)** No differences were observed between sexes about preference for quadrant. **(D)** Males and females of all groups did not show differences for swim speed. **(E)** Males, and females of all groups did not show differences for swim distance. MWM, Morris water maze.

Statistical analyses of the data obtained during the seven consecutive training trials (learning curve) by repeated ANOVA measurements showed an improvement in the performance equally among all animals in search of hidden platform latencies [*F*_(6,726)_ = 252,37; *p* = 0.00] (Figure 6A). When analyzed the interaction there were no differences significant between Days*Groups [*F*_(30,726)_ = 1,0701; *p* = 0.36] (Figure 6A) and Days*Sex [*F*_(6,726)_ = 0.79585; *p* = 0.57]. In the probe trial, one-way Anova did not demonstrate a difference among the groups in relation to the quadrant preference [*F*_(5,121)_ = 0.314; *p* = 0.904] (Figure 6B) and between males and females [*F*_(1,121)_ = 0.058; *P* = 0.810], illustrated in Figure 6C. No significant interaction was observed between Group*Sex [*F*_(5,121)_ = 0.214; *p* = 0.956]. Regarding the latency probe trial to opposite quadrant, no differences were found among the groups [*F*_(5,121)_ = 1.080; *p* = 0.375] (Figure 6B), and between sexes [*F*_(1,121)_ = 0.012; *p* = 0.912] (Figure 6C), nor an interaction between Group*Sex [*F*_(5,121)_ = 1.274; *p* = 0.280]. For the latency to right quadrant variable, there were no differences among the groups [*F*_(5,121)_ = 1.651; *p* = 0.152] (Figure 6B) and between females and males [*F*_(1,121)_ = 0.050; *p* = 0.823] (Figure 6C), nor an interaction between Group*Sex [*F*_(5,121)_ = 0.321; *p* = 0.899]. For the latency to left quadrant variable, no differences among the groups [*F*_(5,121)_ = 0.583; *p* = 0.713] (Figure 6B), between sexes [*F*_(1,121)_ = 0.139; *p* = 0.710] (Figure 6C) and an interaction between Group*Sex [*F*_(5,121)_ = 0.381; *p* = 0.861] were detected. In regards the swim speed, no differences were observed among the groups [*F*_(5,121)_ = 0.748; *p* = 0.589] (Figure 6D), between sexes [*F*_(1,121)_ = 1.165; *p* = 0.283] (Figure 6D) nor an interaction between Group*Sex [*F*_(5,121)_ = 0.541; *p* = 0.745]. For the swim distance variable, no significant differences were detected among the groups [*F*_(5,121)_ = 0.807; *p* = 0.547] (Figure 6E), between females and males [*F*_(1,121)_ = 1.086; *p* = 0.300] (Figure 6E), nor an interaction between Group*Sex [*F*_(5,121)_ = 0.408; *p* = 0.842].

#### Body/brain weights

After the neonatal period (P9), body weight gain was slower in the CFA + F and F groups, although a difference among the groups was not identified in adulthood (P89), as shown in Table 1. A main effect of sex was detected, where the males showed more body mass than females on P89 (*p* < 0.001). No differences in brain and cerebellum mass were found among the experimental groups or between the sexes.

**TABLE 1:** Body mass (in grams) of male and female rats over the course of the study on P1, P9, and P89, according to the experimental groups.

Treatment	Female P1	Female P9	Female P89	Male P1	Male P9	Male P89
Naive	6.4 ± 0.6	16.4 ± 1.4	217.5 ± 12.8	6.4 ± 0.7	17.1 ± 2.0	318.5 ± 40.3
Sham 1	6.0 ± 0.6	16.5 ± 2.0	221.0 ± 20.3	6.2 ± 0.8	16.4 ± 1.2	330.1 ± 33.8
Sham 2	6.0 ± 0.2	16.5 ± 1.0	217.7 ± 20.9	6.1 ± 0.7	16.4 ± 2.0	325.7 ± 25.8
CFA	6.2 ± 0.4	15.8 ± 1.5	211.0 ± 17.5	6.6 ± 0.5	16.5 ± 2.3	335.1 ± 27.1
CFA F	6.4 ± 0.4	14.5 ± 1.3	217.1 ± 8.8	6.4 ± 0.9	15.6 ± 1.2	318.7 ± 28.1
F	6.0 ± 0.3	14.4 ± 1.4	216.3 ± 16.5	6.5 ± 1.0	14.7 ± 1.8	309.5 ± 32.1

## Discussion

In this study, we found age-dependent differences in hippocampal neurogenesis and sex-specific differences in anxiety behavior in adulthood, although all animals presented similar memory retention in adulthood. The literature has shown that both neonatal pain and neonatal exposure to opiates may modulate the functioning of the developing brain (Vinall and Grunau, 2014).

Adult hippocampal neurogenesis is a process involving the continuous generation of newborn neurons in the hippocampus (Altman, 1962). Newly born cells in the dentate gyrus may have diverse ends. Some differentiate into neurons or glia, whereas others may die (Christie and Cameron, 2006). Choosing the right time of BrdU administration regarding the treatment and timing of the animal sacrifice can be complex to assess cell proliferation, cell survival, cell differentiation, and cell death (Christie and Cameron, 2006). In addition, the literature has demonstrated that this process is downregulated by stressful events, such as pain (Duric and McCarson, 2006) and different types of stress in adult rodents (Gould et al., 1997; Pham et al., 2003; Dong et al., 2004). We found increased hippocampal neurogenesis on P10 in rats exposed to an inflammatory nociceptive stimulus on the first day of life with fentanyl pretreatment; however, this result did not persist on P39, which is equivalent to early adolescence in humans. It is important to highlight that most of the studies were performed on adult animals. In our study, the manipulation occurred during the first week of life, a period when the CNS is functionally immature (Romijn et al., 1991). To illustrate this immaturity, there is a significant cortical GABAergic neuron depolarization during the first two weeks of life, which does not persist after this period, when the GABAergic receptor function becomes hyperpolarizing (Owens and Kriegstein, 2002; Ben-Ari et al., 2012).

The premature brain is susceptible to damage, and traumatic experiences are associated with abnormal brain development (Vinall and Grunau, 2014). The hippocampus is a brain region vulnerable to stress throughout life, and repetitive and untreated neonatal pain can disrupt the developing brain of preterm infants in the NICU (Ranger and Grunau, 2014). We aimed specifically to investigate the influence of neonatal pain, with or without fentanyl pretreatment, on the DG of the hippocampus. Duhrsen et al. (2013) reported cell death in rats exposed to mild and severe pain and a relationship between the severity of pain and neuronal injury (Duhrsen et al., 2013).

Pharmacological approaches for pain management have been used in the NICU, and opioids are considered the “gold standard” medication for moderate to severe neonatal pain treatment since they have shown significant efficacy in relieving acute pain in premature infants (Anand, 2001). Although opiates are known to improve behavioral measures in mechanically ventilated infants (Anand, 2001), concerns regarding the impact of neonatal usage of these drugs on the brain have arisen over the last decade. Data reported by Bajic et al. (2013) showed that repeated morphine administration in neonatal rats increased supraspinal apoptosis in the cortex and amygdala, although the hippocampus was not affected (Bajic et al., 2013). One study performed by Craig and Bajic revealed that prolonged morphine exposure in the neonatal period led to thermal hyperalgesia in adulthood (Craig and Bajic, 2015). Another study performed in adult rats demonstrated that chronic exposure to morphine treatment decreased the BrdU-positive cells in the granule layer of the dentate gyrus by 28% when compared to control animals (Eisch et al., 2000). Although the literature has demonstrated the negative modulation of opioids on adult hippocampal neurogenesis (Bortolotto and Grilli, 2017), the impact of neonatal exposure to opioids on hippocampal neurogenesis is still unclear.

The involvement of the hypothalamic/pituitary/adrenal (HPA) axis has been suggested following pain exposure, as the stress response may play a role as a potential mediator of the long-term effects of pain (Sternberg and Al-Chaer, 2007). Although most studies have focused on maternal separation during the neonatal period to evaluate the activation of the HPA axis, noxious stimuli are considered inherent stressors (Schwaller and Fitzgerald, 2014). Neonatal stress may alter the short and long-term functioning of the brain (Meaney et al., 1988), leading to neuroendocrine and behavioral responses, and subsequently could be associated with anxiety-like behaviors in adulthood (Schwaller and Fitzgerald, 2014).

The EPM, one of the most-employed animal models of anxiety for decades, is based on the natural tendency of rodents to explore novel environments and their innate avoidance of unprotected, bright, and elevated places (Campos et al., 2013). In this study, animals exposed to an inflammatory nociceptive stimulus and pretreated with fentanyl showed more anxious behavior, although the groups showed no differences in the OF test. Although we attempted to treat the noxious stimulus initiated on the first day of life, this group of animals was also exposed to many manipulations, including the intraplantar injection of CFA on P1 and eight subcutaneous injections from P1 to P8.

Maternal care for pups experiencing pain has been suggested to reduce offspring pain sensitivity (de Medeiros et al., 2009). In this study, we used a Sharpie pen to mark the pups, since animals of different groups were housed in the same cage. Because of the excessive maternal licking due to the ink, the pups had to be manipulated twice a day for re-marking. This early manipulation could have contributed to blunting the stressful experience initiated on the first day of life. Data reported by Johnston and Walker (2003) showed that pups exposed to nociceptive stimuli during the neonatal period received significantly more grooming from their mothers than pups that only underwent handling (Johnston and Walker, 2003).

The involvement of continuous generation of adult-born neurons in hippocampus-dependent learning and memory of new information has been described by multiple studies (Gould et al., 1999; Leuner et al., 2006). Our results demonstrated increased hippocampal neurogenesis in the animals that were exposed to an inflammatory nociceptive stimulus on the first day of life; however, this finding did not persist in adolescence on P39. Subsequently, no impairment of memory retention was found in any of the groups. In the MWM, adult rats demonstrated a clear reduction in latency equally over seven days and showed no differences in quadrant preference in the probe trial.

Because we exposed the animals to fentanyl during the first week of life and were interested in evaluating the long-term behavioral effects of this early administration, body mass gain assessment was important as a measure of the health condition of the pups (Craig and Bajic, 2015). Our results revealed slower body mass gain among the pups that received fentanyl (F and CFA + F groups) immediately after the neonatal injections, on P9, similar to the data obtained by Craig and Bajic (2015). However, no differences were found among the groups in adulthood, indicating a short-term effect of fentanyl due to increased sleepiness.

Clinical and experimental studies in rodents suggest that males and females respond to pain differently (Unruh, 1996; Berkley, 1997; Lima et al., 2014; Amaral et al., 2015). Neonatal inflammatory injury has a sexually dimorphic response, where females show significantly greater basal hypoalgesia (LaPrairie and Murphy, 2010). Animal and clinical studies have also demonstrated sex-related differences in pain response. Here, we investigated the possible influence of sex on neurogenesis, anxiety behavior, and memory retention in adulthood. Our results suggested more anxious behavior in females, represented by the time spent in the outer area in an OF apparatus. However, we did not find influence of the estrous cycle on the behavioral parameters. Data reported by LaPrairie and Murphy (2007) revealed that females exhibited a significantly greater response to pain, where neonatal injury resulted in significantly greater basal hypoalgesia in adulthood (LaPrairie and Murphy, 2007). Another study performed by Lima et al. (2014) showed an increased hippocampal proliferation cell rate in males, which was directly related to hippocampal brain-derived neurotrophic factor (BDNF) levels (Lima et al., 2014). In addition, the literature has strongly suggested the role of gonadal hormones on neurogenesis modulation, where estradiol may have an effect on cell proliferation in the DG (Galea et al., 2006), which could explain the differences in the cell proliferation on P10 between male and female animals.

Our study had some important limitations. Assessment of hippocampal cell death was difficult despite our efforts using Tunel and Fluor-Jade techniques. The choice of the most suitable nociceptive paradigm in an animal model that resembles the neonatal intensive care unit scenario may be challenging, since there are different animal models of neonatal injury in early life (Schwaller and Fitzgerald, 2014). In the current study, we selected the prolonged inflammatory nociceptive paradigm, based on our previous experience with this animal model (Leslie et al., 2011). In addition, we limited our area of interest to the dentate gyrus. Exploring other areas, such as CA1 and hilus could have added more information about the hippocampal neurogenesis on P39.

In conclusion, our data revealed differences in hippocampal neurogenesis and anxiety behavior when the noxious stimulus was associated with analgesia during the first week of life. Although pain and exposure to opiates have been identified as modulators in the adult brain, the mechanisms and extension of their effects on the CNS are not completely elucidated in a critical period of brain development, such as in neonatal rats.

## Data availability statement

The raw data supporting the conclusions of this article will be made available by the authors, without undue reservation.

## Ethics statement

The animal study was reviewed and approved by Comissão de Ética no Uso de Animais (CEUA), Universidade Federal de São Paulo.

## Author contributions

DR performed all of the animal manipulations and the statistical analysis and contributed to the manuscript elaboration. CS supported the immunohistochemistry protocols and assisted in the elaboration of the manuscript. LM contributed to the interpretation of the results and assistance in the elaboration of the manuscript and its critical assessment. AL contributed to the study design and writing and revising the manuscript. The work presented here was done in collaboration with all authors. All authors reviewed and approved the final manuscript.

## Supplementary material

Supplementary Table 1 Estrous cycle and behavioral outcomes.
